# Altered thyroid hormone profile in offspring after exposure to high estradiol environment during the first trimester of pregnancy: a cross-sectional study

**DOI:** 10.1186/s12916-014-0240-0

**Published:** 2014-12-16

**Authors:** Ping-Ping Lv, Ye Meng, Min Lv, Chun Feng, Ye Liu, Jing-Yi Li, Dan-Qin Yu, Yan Shen, Xiao-Lin Hu, Qian Gao, Shan Dong, Xian-Hua Lin, Gu-Feng Xu, Shen Tian, Dan Zhang, Fang-Hong Zhang, Jie-Xue Pan, Xiao-Qun Ye, Miao-E Liu, Xin-Mei Liu, Jian-Zhong Sheng, Guo-Lian Ding, He-Feng Huang

**Affiliations:** Key Laboratory of Reproductive Genetics, Ministry of Education, Zhejiang University, 388 Yuhangtang Road, Hangzhou, Zhejiang 310058 China; International Peace Maternity and Child Health Hospital, Shanghai Jiao Tong University School of Medicine, 910 Hengshan Road, Shanghai, 200030 China

**Keywords:** Early pregnancy, Embryo transfer, Estradiol, Intrauterine environment, *In vitro* fertilization, Offspring, Thyroid hormone, Thyroid-stimulating hormone

## Abstract

**Background:**

The increasing number of babies conceived by *in vitro* fertilization and embryo transfer (IVF-ET) shifts concern from pregnancy outcomes to long-time health of offspring. Maternal high estradiol (E_2_) is a major characteristic of IVF-ET and lasts throughout the first trimester of pregnancy. The fetal thyroid develops during this period and may thus be affected by exposure to the supra-physiological E_2_. The aim of this study is to investigate whether the high E_2_ maternal environment in the first trimester increases the risk of thyroid dysfunction in children born following IVF-ET.

**Methods:**

A cross-sectional survey design was used to carry out face-to-face interviews with consecutive children attending the hospital. A total of 949 singletons born after fresh embryo transfer (ET) (n = 357), frozen ET (n = 212), and natural conception (NC) (n = 380), aged 3 to 10 years old, were included. All children were thoroughly examined. Meanwhile, another 183 newborns, including 55 fresh ET, 48 frozen ET, and 80 NC were studied. Levels of serum T3, FT3, T4, FT4, and TSH and levels of maternal E_2_ at different stages of the first trimester were examined.

**Results:**

The mean serum E_2_ levels of women undergoing fresh ET during the first trimester of pregnancy were significantly higher than those of the women undergoing frozen ET or following NC. The thyroid hormone profile, especially the levels of T4, FT4, and TSH, were significantly increased in 3- to 10-year-old children conceived by fresh ET compared to NC. The same tendency was confirmed in newborns. However, levels of T4 and TSH in the frozen ET group were nearer to that of the NC group. Furthermore, levels of T4 and FT4 in fresh ET were positively correlated with maternal serum levels of E_2_ during early pregnancy.

**Conclusions:**

The maternal high E_2_ environment in the first trimester is correlated with increased risk of thyroid dysfunction. Frozen ET could reduce risks of thyroid damage in children conceived by IVF. Further studies are needed to confirm these findings and to better determine the underlying molecular mechanisms and clinical significance.

**Trial registration:**

ChicCTR-OCC-14004682 (22-05-2014)

## Background

Assisted reproduction technology (ART) has been applied for treating infertility for more than three decades since the first successful case by Steptoe in 1978 [1]. Up to now, there are over five million children in the world who were born following *in vitro* fertilization (IVF) and embryo transfer (ET). The increasing number of IVF babies shifts concern from pregnancy outcomes to the long-term health of IVF offspring. It is reported that babies conceived through ART are at increased risks of low birth weight and being small for their gestational age, which are themselves associated with increased risks of adult diseases including diabetes mellitus and cardiovascular diseases [2-6]. However, observational studies have not shown any significant difference in neuron developmental outcomes with conflicting results regarding the presence of cerebral palsy [7]. Interestingly, thyroid function studies in IVF children have been quite limited. The early occurrence of thyroid dysfunction, if not promptly treated, causes serious and irreversible damage to the central nervous system with subsequent mental impairment and may predispose to cardiovascular disease in the long-term [8,9]. Additionally, emerging studies suggest that the pre-implantation period is vulnerable to epigenetic perturbations and call for systematic long-term follow-up of IVF children [10].

In fresh ET cycles, controlled ovarian hyperstimulation (COH) of ART situates the gamete/embryo in a supra physiological estradiol (E_2_) environment, and there is further evidence that E_2_ may influence thyroid functions [11-14]. In our previous study, we found that increased E_2_ concentrations after COH not only appeared before and during implantation but also after implantation; this is an effect which could last throughout the first trimester [3]. Since the abnormal endocrine environment of increased E_2_ after COH could persist to first trimester of pregnancy, fetal development during this period may be impaired. This is likely to be the case for hormone-sensitive organs such as the thyroid gland which is the first endocrine gland to differentiate in the early embryo at approximately 3 to 5 weeks gestation [15]. As one of the largest endocrine glands, the primary function of the thyroid gland is to synthesize thyroid hormones by metabolizing iodide. Thyroid hormones are critical regulators of growth, development, and metabolism in almost all tissues. However, few studies have investigated the thyroid function of children conceived through IVF and therefore long-term monitoring of their thyroidal status is needed [16].

The aim of this study is to investigate possible thyroid dysfunction in fresh ET children compared with natural conception (NC) and to evaluate whether the high E_2_ environment during early pregnancy was causative. Because frozen ET is performed in the natural cycle with a normal intrauterine environment, we studied 212 children conceived after frozen ET and investigated whether avoiding the exposure to high E_2_ environment during the first trimester could reduce the risk of thyroid dysfunction. Additionally, we collected umbilical cord blood of newborns conceived by fresh ET, frozen ET, and NC and analyzed whether the levels of thyroid hormones correlated with maternal serum E_2_ levels during early pregnancy in the fresh ET group.

## Methods

### Study cohort

A cross-sectional survey was undertaken at the ART Unit in the Women’s Hospital, School of Medicine, Zhejiang University, China. The study was approved by the Ethical Committee of Women’s Hospital, School of Medicine, Zhejiang University and was registered in the Chinese Clinical Trial Registry (ChicCTR-OCC-14004682). The ART unit is one of the earliest licensed by the Health Ministry of People’s Republic of China and is also one of the largest in the country. A total of 949 Asian singletons aged 3 to 10 years old delivered from January 2003 to March 2011 were included in this study of which 357 cases were after fresh ET, 212 cases after frozen ET, and 380 after NC. Face-to-face interviews were carried out and the children were examined thoroughly. Interviews took place in the visit room individually and lasted approximately 30 minutes. Written consent was obtained in every case prior to assessment.

All NC cases were randomly selected and matched for age and gender to cases in the fresh ET group and frozen ET group. These patients had been referred to the Physical Examination Center for a regular health examination and were recruited when they agreed to participate. All cases were healthy, did not take any medication, and lived in iodine-replete areas. None of the NCs had congenital hypothyroidism. Mothers with thyroiditis, hyperthyroidism, or hypothyroidism during pregnancy were excluded.

Additionally, we studied 183 full-term singleton infants, including 55 fresh ET, 48 frozen ET, and 80 NC born in our hospital. They were matched according to maternal age, gestational age, and birth weight and all IVF babies were conceived after traditional IVF, whereas infants conceived after intracytoplasmic sperm injection were excluded. All newborns selected above were free of obstetric complications during pregnancy such as pregnancy-induced hypertension, gestational diabetes mellitus, placenta previa, and intrahepatic cholestasis. Mothers with thyroiditis, hyperthyroidism, or hypothyroidism during pregnancy were also excluded.

During all the fresh ET cycles, ovarian stimulations were performed by the standard long or short protocols as previously described [17]. Oocyte retrieval was carried out 34 to 36 h after hCG injection. Fresh and frozen ET were performed on Day 2 or Day 3. Cryopreserved embryos were thawed on the day of the transfer and all the frozen ETs proceeded in natural cycles without COH. Children and babies from the fresh or frozen ET groups were excluded if the women had received donated oocytes or sperm, or if they had undergone pre-implantation genetic diagnosis.

On the other hand, maternal serum E_2_ levels on hCG administration day of 55 fresh ET newborns were obtained from database of the hospital. In order to detect the maternal E_2_ level, blood samples of another 530 women with singleton conceptions at 4 to 12 weeks of gestation were obtained from October 2012 to June 2013, including 206 fresh ET, 124 frozen ET, and 200 NC and their clinical gestational data were also obtained from the database.

### History records and physical examination

A detailed medical history was obtained from all participants. Data recorded included the maternal age, sex, ART methods, infant birth weight, birth length, gestational age, and mode of feeding. Gestational age was calculated based from the date of ET and the date of birth. The same trained female nurse examined all children. Physical examination included height, weight, body mass index, systolic blood pressure, diastolic blood pressure, and heart rate. We had access to complete clinical data for each individual.

### Serum levels of thyroid hormones in children and newborns

Samples were collected from umbilical cord blood of newborns at birth and peripheral blood of 3- to 10-year-old children at 8:00 am after overnight fasting. Serum T3, FT3, T4, FT4, and TSH were assayed with the Abbott Architect i2000 assay (Abbot Diagnostics) in the local laboratory. The intra- and inter-assay coefficients of variation for the determination of all biochemical variables were less than 5%.

### Correlation analysis of maternal serum E_2_ levels with thyroid hormones of offspring

Characteristics and cycle parameters of patients were obtained from databases of the Department of Reproductive Endocrinology, Women’s Hospital, School of Medicine, Zhejiang University. To explore the correlation between maternal E_2_ level and thyroid hormones in offspring, we analyzed serum levels of T4 and FT4 of 55 fresh ET newborns and gestational E_2_ levels on the day that their mothers received hCG.

### Statistical analysis

Either the two-tailed Student’s *t*-test or ANOVA were used to evaluate continuous parametric data. Relationships between type of conception and thyroid function was analyzed by a regression model with adjustment for age of child, type of assisted reproduction, and sex of child or maternal variables (pregnancy-induced hypertension, gestational diabetes mellitus, placenta previa, or intrahepatic cholestasis); the χ^2^ test was used to compare categorical data. Pearson’s correlation coefficient was applied to explore the association of T4 and FT4 with E_2_ levels. Log transformations were conducted when the normality assumption was not satisfied. Analyses were conducted using SPSS statistical software (version 16.0) and *P* <0.05 indicated statistical significance.

## Results

### Characteristics

Table 1 presents physical examination and medical history characteristics of children conceived by fresh ET, frozen ET, and NC. All groups were similar according to age, gender, birth length, mode of feeding, current weight/length, rate of preterm delivery, blood pressure, heart rate, and maternal age. Children conceived by fresh ET had significantly lower gestational age and birth weight compared with NC, whereas no statistically significant differences were observed between frozen ET and NC. Maternal age in the NC group was significantly lower than in the fresh ET and frozen ET groups. Table 2 presents the physical examination and medical history characteristics of newborns born after fresh ET, frozen ET, and NC. All groups were similar according to gender, birth weight/length, gestational age, and maternal age.

**Table 1:** **Physical examination characteristics and medical history of children conceived by natural conception (NC), fresh embryo transfer (ET), and frozen ET**

		**NC**	**Fresh ET**	**Frozen ET**	***P*** **value**	***P*** **value**	***P*** **value**
		**(n = 380)**	**(n = 357)**	**(n = 212)**	**(Fresh ET vs. NC)**	**(Fresh ET vs. Frozen ET)**	**(Frozen ET vs. NC)**
**Age (yr)**		4.98 ± 1.47	5.01 ± 1.12	4.96 ± 1.01	NS	NS	NS
**Gender, n (%)**	Boy	194 (51.1%)	186 (52.1%)	95 (44.8%)	NS	NS	NS
	Girl	186 (48.9%)	171 (47.9%)	117 (55.2%)			
**Birth weight (g)**		3376 ± 381	3307 ± 526	3322 ± 553	<0.05	NS	NS
**Birth length (cm)**		50.07 ± 0.95	49.93 ± 2.27	49.80 ± 2.70	NS	NS	NS
**Gestational age (wk)**		38.76 ± 1.63	38.32 ± 1.70	38.48 ± 1.58	<0.01	NS	<0.05
**BMI**		15.49 ± 1.44	15.28 ± 1.68	15.47 ± 1.72	NS	NS	NS
**Weight (kg)**		19.29 ± 3.57	19.22 ± 4.26	19.03 ± 4.03	NS	NS	NS
**Length (cm)**		111.19 ± 8.47	111.67 ± 8.25	110.28 ± 8.66	NS	NS	NS
**Preterm delivery**	No	350 (92.1%)	318 (89.1%)	187 (88.2%)	NS	NS	NS
**(<37 wk), n (%)**	Yes	30 (7.9%)	39 (10.9%)	25 (11.8%)			
**ART method**	IVF	/	252 (70.6%)	152 (71.7%)	NA	NS	NA
	ICSI	/	105 (29.4%)	60 (28.3%)			
**Mode of feeding**	Breast	207 (54.4%)	197 (55.2%)	115 (54.2%)	NS	NS	NS
	Artificial	80 (21.1%)	66 (18.5%)	52 (24.5%)			
	Mixed	93 (24.5%)	94 (26.3%)	45 (21.3%)			
**Blood pressure (mmHg)**	SBP	97.86 ± 14.09	97.71 ± 16.19	97.35 ± 16.59	NS	NS	NS
	DBP	56.56 ± 9.05	55.77 ± 11.42	56.35 ± 11.38	NS	NS	NS
**Heart rate (bpm)**		96.94 ± 12.52	96.26 ± 13.76	97.44 ± 12.64	NS	NS	NS
**Maternal age (yr)**		29.69 ± 3.63	31.05 ± 3.70	31.35 ± 3.66	<0.01	NS	<0.01
**Pregnancy complications, n (%)**							
**Gestational diabetes**		12 (3.2%)	12 (3.4%)	11 (5.2%)	NS	NS	NS
**Intrahepatic cholestasis**		8 (2.1%)	5 (1.4%)	2 (0.9%)	NS	NS	NS
**Pre-eclampsia**		8 (2.1%)	13 (3.6%)	3 (1.4%)	NS	NS	NS
**Placenta previa**		14 (3.7%)	12 (3.4%)	5 (2.4%)	NS	NS	NS

Data are presented as mean ± SD or n (%), NS, not significant; NA, Not applicable; ART, Assisted reproduction technology; BMI, Body mass index; DBP, Diastolic blood pressure; IVF, *In vitro* fertilization; ICSI, Intracytoplasmic sperm injection; SBP, Systolic blood pressure.

**Table 2:** **Physical examination characteristics and medical history of newborns conceived by natural conception (NC), fresh embryo transfer (ET), and frozen ET**

		**NC**	**Fresh ET**	**Frozen ET**	***P*** **value**	***P*** **value**	***P*** **value**
		**(n = 80)**	**(n = 55)**	**(n = 48)**	**(Fresh ET vs. NC)**	**(Fresh ET vs. Frozen ET)**	**(Frozen ET vs. NC)**
**Singletons/Twins**		Singletons	Singletons	Singletons	NS	NS	NS
**Gender, n (%)**	Boy	41 (51.3%)	27 (49.1%)	23 (47.9%)	NS	NS	NS
	Girl	39 (48.7%)	28 (50.9%)	25 (52.1%)			
**Birth weight (g)**		3411 ± 305	3385 ± 488	3404 ± 389	NS	NS	NS
**Birth length (cm)**		50.03 ± 0.83	49.72 ± 0.81	49.91 ± 0.72	NS	NS	NS
**Gestational age (wk)**		38.63 ± 0.95	38.37 ± 0.91	38.44 ± 1.04	NS	NS	NS
**Preterm delivery (<37 wk), n (%)**	No	80 (100%)	55 (100%)	48 (100%)	NS	NS	NS
	Yes	0 (0%)	0 (0%)	0 (0%)			
**ART method**	IVF	/	55 (100%)	48 (100%)	NA	NS	NA
	ICSI	/	0 (0%)	0 (0%)			
**Maternal age (yr)**		30.53 ± 3.17	31.31 ± 3.07	31.73 ± 3.83	NS	NS	NS
**Pregnancy complications, n (%)**		0	0	0	NS	NS	NS

Data are presented as mean ± SD or n (%), NS, not significant; NA, Not applicable. ART, Assisted reproduction technology; BMI, Body mass index; IVF, *In vitro* fertilization; ICSI, Intracytoplasmic sperm injection.

### Maternal serum levels of E_2_ during the first trimester after fresh ET, frozen ET, and NC

**Figure 1:**
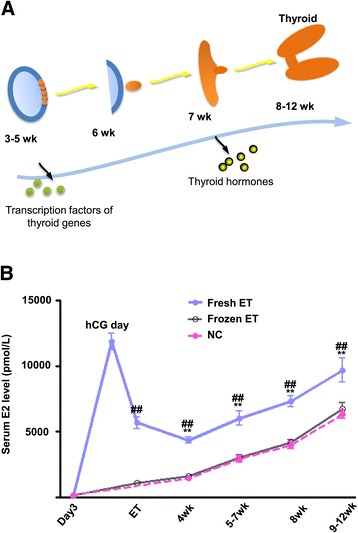
**Schematic view of thyroid morphogenesis and maternal serum estradiol levels during early pregnancy. (A)** Schematic view of thyroid morphogenesis in human development. The thyroid gland begins to develop at 3 to 5 weeks of gestation as an endodermal thickening in the floor of the primitive pharynx. From week 5, thyroid precursor cells express a combination of transcription factors which are required for the early stages of thyroid development. After losing all connections with the pharynx, the thyroid bud migrates caudally, reaching its final position in front of the trachea at 6 weeks. Thyroid follicular cells begin their differentiation program and express thyroid-specific genes and secrete thyroid hormones from week 7. Finally, primitive follicles appear and the gland displays its final morphological organization (8 to 12 weeks) [12]. **(B)** Maternal serum estradiol concentrations during the first trimester of pregnancy. The mean serum levels of estradiol (E_2_) in the fresh embryo transfer (ET) group (n = 206) at different stages were all significantly higher than those in the frozen ET (n = 124) and natural conception (NC) (n = 200) groups during the first trimester of pregnancy. Data are presented as mean ± SEM, **P <0.01 vs. NC, ^##^ *P* <0.01 vs. frozen ET. ET day, day of embryo transfer; hCG day, day of hCG administration.

The most critical period of fetal thyroid development is the first trimester of pregnancy (Figure 1A). We analyzed the maternal serum levels of E_2_ during the first trimester in 206 patients after fresh ET, 124 patients after frozen ET, and 200 after NC (Figure 1B). In addition to the much higher level of E_2_ upon hCG administration, the mean levels of serum E_2_ of mothers in the fresh ET group were 4,351.3 ± 250.4 pmol/L at 4 weeks, 6,027.7 ± 529.7 pmol/L at 5 to 7 weeks, 7,305.2 ± 420.1 pmol/L at 8 weeks, and 9,691.9 ± 903.7 pmol/L at 9 to 12 weeks of gestation, which were all significantly higher than those in the frozen ET group (1,586.2 ± 126.9 pmol/L at 4 weeks, 3,020.3 ± 227.7 pmol/L at 5 to 7 weeks, 4,168.1 ± 238.1 pmol/L at 8 weeks, and 6,748.0 ± 482.2 pmol/L at 9 to 12 weeks, *P* <0.01, respectively), and those in NC group (1,427.9 ± 63.4 pmol/L at 4 weeks, 2,911.5 ± 249.4 pmol/l at 5 to 7 weeks, 3,968.3 ± 151.1 pmol/L at 8 weeks, and 6,310.5 ± 309.7 pmol/L at 9 to 12 weeks, *P* <0.01, respectively, Figure 1B). There was no significant difference between E_2_ levels in the frozen ET and NC groups during the first trimester (Figure 1B).

### Altered thyroid hormone profile of children born after fresh ET compared with frozen ET and NC

We compared the serum levels of thyroid hormone profile between children conceived by fresh ET, frozen ET, and NC. Except for gestational age, birth weight, and maternal age, there were no significant differences in physical examination characteristics and medical history among the NC, fresh ET, and frozen ET groups (Table 1). Interestingly, in 3- to 10-year-old children conceived by fresh ET, the levels of T4, FT4, and TSH were significantly higher than those of children conceived by NC (crude *P* <0.01, Table 3). Although there was no significant difference, the levels of T3 and FT3 tended to be higher in the fresh ET group. The observation that frozen ET can avoid exposure to high maternal E_2_ environment in IVF led us to compare the thyroid hormone profiles between the children conceived by fresh ET and frozen ET. We found that levels of T4 and TSH in the frozen ET group were significantly lower (crude *P* <0.01, Table 3) and nearer to the values of the NC group. In order to solidify the results, we further analyzed the relationship between type of conception and thyroid function with adjustment for age of child, ART type, and sex of child or maternal variables (pregnancy-induced hypertension, gestational diabetes mellitus, placenta previa, or intrahepatic cholestasis) by a regression model adjusting. We found that levels of T4, FT4, and TSH were still significantly higher than those of children conceived by NC (Adjusted *P* <0.01, Table 3). However, the levels of T4 and TSH in the frozen ET group were significantly lower than fresh ET (Adjusted *P* <0.01, Table 3).

**Table 3:** **The levels of thyroid hormone profile in children conceived by natural conception (NC), fresh embryo transfer (ET), and frozen ET**

	**NC**	**Fresh ET**	**Frozen ET**	**Fresh ET vs. NC Crude** ***P***	**Fresh ET vs. Frozen ET Crude** ***P***	**Frozen ET vs. NC Crude** ***P***
	**(n = 380)**	**(n = 357)**	**(n = 212)**	**(Adjusted** ***P*** **)**	**(Adjusted** ***P*** **)**	**(Adjusted** ***P*** **)**
T3 (nmol/L)	2.28 ± 0.22	2.31 ± 0.32	2.31 ± 0.41	0.06 (0.09)	0.78 (0.77)	0.25 (0.32)
FT3 (pmol/L)	5.92 ± 0.61	6.01 ± 0.73	5.98 ± 0.91	0.07 (0.07)	0.65 (0.83)	0.36 (0.20)
T4 (nmol/L)	100.1 ± 17.06	105.81 ± 16.82	100.76 ± 15.75	<0.01 (<0.01)	<0.01 (<0.01)	0.64 (0.91)
FT4 (pmol/L)	15.76 ± 1.67	16.55 ± 1.74	16.46 ± 1.61	<0.01 (<0.01)	0.54 (0.69)	<0.01 (<0.01)
TSH (mIU/L)	2.35 ± 0.96	2.69 ± 1.37	2.41 ± 0.98	<0.01 (<0.01)	<0.01 (<0.01)	0.49 (0.51)

Data are shown as mean ± SD.

Log transformations were conducted when the normality assumption was not satisfied. Adjusted *P* values were calculated by linear regression adjusted for age of child, type of assisted reproduction, sex of child, or maternal variables (obstetric complications).

### Increased risk of thyroid disorders in children conceived by fresh ET

We further calculated and compared the rate of 3- to 10-year-old children whose serum T4, FT4, or TSH levels were beyond the standard values. Two out of 357 children conceived by fresh ET (0.6%) were found to have T4 levels exceeding 150.84 nmol/L, while none was detected in the NC group. FT4 exceeding 19.05 pmol/L was present in 26 of 357 children conceived by fresh ET (7.3%) and in none in the NC group (*P* <0.01, Table 4). TSH exceeding 5.05 mIU/L was present in 22 of 357 children conceived by fresh ET (6.2%) and in 3 of 380 of the NC group (0.08%) (*P* <0.01, Table 4). Further, one child showed both elevated FT4 (>19.05 nmol/L) and TSH (>5.05 mIU/L) in the fresh ET group, whereas none did so in either the frozen ET or NC groups. Interestingly, no significant difference was found between serum TSH levels in the 3- to 10-year-old children conceived by frozen ET and those by NC (2.4% vs. 0.08%). Although the incidence of elevated FT4 in the frozen ET group (4.7%) was still significantly higher than that of the NC group (*P* <0.01, Table 4), compared to fresh ET, a decreased tendency of risk was observed in the frozen ET group.

**Table 4:** **The incidence of 3- to 10-year-old children whose serum level of T4, FT4, or TSH beyond the standard values in natural conception (NC), fresh embryo transfer (ET), and frozen ET groups**

		**NC**	**Fresh ET**	**Frozen ET**	**Fresh ET vs. NC**	**Fresh ET vs. Frozen ET**	**Frozen ET vs. NC**
		**(n = 380)**	**(n = 357)**	**(n = 212)**	***P*** **value**	***P*** **value**	***P*** **value**
T4	>150.84 (nmol/L)	0 (0%)	2 (0.6%)	0 (0%)	0.23	0.53	NA
FT4	>19.05 (pmol/L)	0 (0%)	26 (7.3%)	10 (4.7%)	<0.01	0.29	<0.01
TSH	>5.05 (mIU/L)	3 (0.08%)	22 (6.2%)	5 (2.4%)	<0.01	0.04	0.14

Data are shown as n (%). NA, Not applicable.

### Umbilical levels of thyroid hormones and TSH in newborns born after fresh ET and NC

Additionally, we examined the levels of T4, FT4, T3, FT3, and TSH in umbilical blood in 183 newborns, including 55 fresh ET cases, 48 frozen ET cases, and 80 NC cases. There were no significant differences in physical examination characteristics and medical history among the three groups (Table 2). Surprisingly, in newborns conceived by fresh ET, the levels of T4, FT4, and TSH were significantly higher than those of children conceived by NC (crude *P* <0.01, *P* <0.05, and *P* <0.01, respectively, Table 5). However, the levels of T4 and TSH in the frozen ET group were significantly lower than in the fresh ET group (crude *P* <0.05, Table 5), which were similar to values in the NC group. No significant differences in the levels of T3 and FT3 were found among the three groups (crude *P* >0.05, respectively). Moreover, we further analyzed the relationship between type of conception and thyroid function with adjustment for sex. We found that levels of T4, FT4, and TSH were also significantly higher than those in newborns conceived by NC (Adjusted *P* <0.01, *P* <0.05, and *P* <0.05, respectively, Table 5). However, levels of T4 and TSH in the frozen ET group were significantly lower compared with fresh ET (Adjusted *P* <0.05, Table 5). There was no significant difference between frozen ET and NC.

**Table 5:** **The levels of thyroid hormone profiles in newborns conceived by natural conception (NC), fresh embryo transfer (ET), and frozen ET**

	**NC**	**Fresh ET**	**Frozen ET**	**Fresh ET vs. NC**	**Fresh ET vs. Frozen ET**	**Frozen ET vs. NC**
	**(n = 80)**	**(n = 55)**	**(n = 48)**	**Crude** ***P***	**Crude** ***P***	**Crude** ***P***
				**(Adjusted** ***P*** **)**	**(Adjusted** ***P*** **)**	**(Adjusted** ***P*** **)**
T3 (nmol/L)	2.09 ± 0.44	2.13 ± 0.47	1.94 ± 0.65	0.60 (0.66)	0.09 (0.09)	0.18 (0.10)
FT3 (pmol/L)	4.93 ± 0.92	5.07 ± 1.25	5.02 ± 1.57	0.43 (0.43)	0.85 (0.84)	0.52 (0.67)
T4 (nmol/L)	98.41 ± 14.71	107.65 ± 18.93	99.69 ± 21.43	<0.01 (<0.01)	0.03 (0.03)	0.69 (0.73)
FT4 (pmol/L)	15.66 ± 1.97	16.50 ± 1.76	16.39 ± 2.47	0.01 (0.01)	0.82 (0.82)	0.04 (0.07)
TSH (mIU/L)	2.69 ± 0.99	3.15 ± 1.01	2.76 ± 1.00	<0.01 (0.01)	0.03 (0.03)	0.91 (0.78)

Data are shown as mean ± SD.

Log transformations were conducted when the normality assumption was not satisfied. Adjusted *P* values were calculated by linear regression adjusted for sex of newborn.

### Maternal serum E_2_ levels on the hCG administration day positively correlated with levels of T4/FT4 in newborns conceived by fresh ET

**Figure 2:**
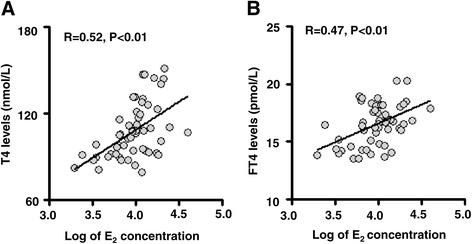
**Correlation between maternal serum E**_**2**_ **levels at HCG day and T4/FT4 levels in fresh embryo transfer (ET) offspring.** The log of maternal serum E_2_ concentrations on the hCG administration day positively correlated with the serum levels of **(A)** T4 and **(B)** FT4 in newborns conceived by fresh ET (r = 0.52, *P* <0.01, n = 55 and r = 0.35, *P* <0.01, n = 55, respectively).

To further explore the relationship between maternal serum E_2_ levels and the thyroid hormone profile, a total cohort of 55 newborn singletons born after fresh ET and their mothers were investigated further. We examined the serum E_2_ levels on the day of hCG administration in these patients. We adopted bivariate correlation analysis to investigate the correlation between thyroid hormones and E_2_, and found that the maternal serum E_2_ levels on the day of hCG administration positively correlated with levels of T4 and FT4 of their offspring (r = 0.52, *P* < 0.01 and r = 0.47, *P* < 0.01, respectively; Figure 2A,B).

## Discussion

Evidence has indicated that an adverse intrauterine environment influences fetal growth and health [3-6,18]. Maternal undernutrition during pregnancy could induce low birth weight, impaired glucose tolerance, and obesity in offspring [19-21]. Intrauterine exposure to diabetes is associated with increased risk of abnormal glucose homeostasis in offspring beyond that attributable to genetic factors [22,23]. In a previous study, we found that a high E_2_ environment is correlated with an increased risk of low birth weight and small-for-gestational age size, which may lead to chronic diseases in later life. COH results in increased E_2_ concentrations not only before and during implantation, but also after implantation, which lasts throughout the first trimester [3]. It is suggested that besides birth weight, the abnormal maternal endocrine environment may also affect the fetal system developed during the first trimester after fresh ET. In our study, we analyzed maternal serum levels of E_2_ at different stages during the first trimester and found the maintenance of a high level of E_2_ in the fresh ET group. In contrast, the level of E_2_ in the frozen ET group was similar to that of the NC group.

The thyroid is one of the earliest endocrine glands to develop in the fetus [15,24] and E_2_ may influence thyroid functions [11,13,14]. In our present work, we investigated the thyroid hormone profile of 3- to 10-year-old singletons conceived by fresh ET, frozen ET, and NC. We found that the levels of T4 and FT4 were both significantly higher in the fresh ET group than in the NC and frozen ET groups. The levels of T3 and FT3 also showed an increased tendency in the fresh ET group, although with no significant difference. Moreover, the percentage of 3- to 10-year-old children whose serum FT4 or TSH levels were beyond the standard value were significantly increased in the fresh ET group, demonstrating the increased risk of thyroid disease in children conceived by fresh ET. According to the hypothalamus-pituitary-thyroid (HPT) feedback loop, elevated T4/FT4 is always accompanied by suppressed TSH [25]. However, at the same time, compared to NC, we found that both TSH and T4/FT4 concentrations increased significantly in the fresh ET group, probably since, in 3- to 10-year-old children, the HTP axis is gradually establishing and improving, and the feedback mechanism is not an exact response [26]. Actually, some children showed elevated T4 and/or FT4 and some showed elevated TSH alone, whereas some showed elevated FT4 and TSH. Except for an immature HTP axis, since early pregnancy is also an important period for neural system development [27,28], the high E_2_ environment maybe also attenuate hypothalamus and/or pituitary functions, inducing a disordered hormone secretion.

The IVF-ET is a complicated process including COH, IVF, *in vitro* culture, and related processes. The most critical difference between fresh ET and frozen ET is the high E_2_ environment during early pregnancy. As expected, the incidence of children with elevated TSH in the frozen ET group was significantly lower than that of the fresh ET group, indicating that the risk was obviously reduced when the high estradiol environment during early pregnancy was relieved. However, FT4 in the frozen ET group ranged between NC and fresh ET groups. The explanation may be that, during IVF treatment, in addition to the intrauterine development, gametes and zygotes/embryos are also exposed to a series of non-physiological processes, especially a high E_2_ environment and culture media [29-31]. The adverse effects of IVF treatment on germ lines and embryos may also cause fetal thyroid damage.

In order to explore whether intrauterine exposure to a high E_2_ environment could alter the thyroid function of offspring as early as birth, we further examined the thyroid hormone profile in newborns of fresh ET, frozen ET, and NC. There was no significant difference in birth weight and other physical characteristics among three groups. It is noteworthy that the significantly elevated levels of T4, FT4, and TSH not only existed in 3- to 10-year-old children, but were also observed in newborns. Previously, we have confirmed the serum E_2_ level on the hCG administration day is an effective marker reflecting the E_2_ level during early pregnancy [3]. We analyzed the association between maternal E_2_ at hCG day and T4/FT4 in newborns conceived by fresh ET, finding a positive correlation. The results demonstrated that early exposure to a high maternal E_2_ environment is directly responsible for hyperactive fetal thyroid function, which persists throughout childhood.

Sakka et al. studied a total of 106 children conceived after classic IVF and 68 naturally conceived controls, aged 4 to 14 years old, and found that 7 IVF children but none of the controls had persistent elevations of circulating TSH. They also found the level of TSH was significantly higher in the IVF group than in controls, which is consistent with our results, whereas no significant differences in the concentrations of T3 or T4 were observed in their study [16]. In our study, we enlarged the number and added the detection of frozen ET children as well as newborns, indicating that the thyroid hormone profile altered as early as birth and that frozen ET could reduce the risks of thyroid damage in children conceived by ET due to a normal intrauterine environment.

Thyroid hormones play an important role in regulating lipid metabolism and thyroid dysfunction can result in lipid abnormalities which increase the risk of hypertension, cardiovascular disease, and endothelial dysfunction [32]. The increased cardiovascular risk in thyroid dysfunction is related to lipid profile, endothelial dysfunction, metabolic, hormonal, and hemodynamic changes, and coagulation disturbances [9]. Caraccio et al. reported that subclinical hypothyroidism adversely affected some surrogate markers for cardiovascular disease [33]. Moreover, a previous study reported that mental changes were always accompanied with thyroid dysfunction, perhaps due to stimulation by the protean actions of the thyroid hormone and thyroid gland playing a role in the pathogenesis of psychiatric disorders [8]. Accumulating studies showed that IVF children manifested significant increases of arterial blood pressure and adipose tissue distribution as well as a higher level of fasting blood glucose [34,35]. In addition, recent studies in IVF-conceived mice have displayed increased fasting glucose and impaired glucose tolerance [2]. These cardiometabolic alterations and lipid metabolic disorder in IVF children might be partly attributed to a higher occurrence of thyroid dysfunction. Therefore, early occurrence of thyroid dysfunction, if not promptly treated, will cause serious and irreversible damage to the cardiovascular system and predisposes to dyslipidemia, mental changes, and metabolic disorders later in life.

A plausible explanation for the abnormal thyroid hormone profile among children conceived by fresh ET is the possible epigenetic alterations that might occur during early pregnancy after exposure to a high E_2_ environment. There is now substantial evidence suggesting that ART pregnancies are associated with altered outcomes in fetal and neonatal development, mainly due to epigenetic modifications of gene expression [36-39]. The fetal thyroid develops during the first trimester of pregnancy and may be affected by exposure to supra-physiological E_2_. Some previous reports have indicated that E_2_ might regulate thyroid function through a direct action on the thyrocytes [40-42]. Antico-Arciuch et al. proved that E_2_ is directly responsible for the increased susceptibility to thyroid disease on activation of the PI3K pathway [43]. Additionally, E_2_ effects on thyroid iodide uptake and thyroperoxidase activity have been confirmed in animal studies [44]. Ho et al. found permanent alterations in the DNA methylation patterns of multiple cell signaling genes after transient developmental exposure to E_2_, suggesting an epigenetic basis for estrogen imprinting [45]. Thus, the altered thyroid hormone profile observed in fresh ET offspring, may be the result of epigenetic alteration in the set point of thyroid hormone sensitivity induced by the high E_2_ environment.

## Conclusions

Our results demonstrate that T4, FT4, and TSH levels were significantly increased in newborns as well as in children aged 3 to 10 years old conceived by fresh ET compared to NC. A remarkably higher maternal E_2_ level was maintained in the fresh ET group during early pregnancy and was positively correlated with the increased levels of T4 and FT4. Frozen-thawed ET, conducted in the normal conception cycles to avoid the high E_2_ environment during the first trimester, could reduce the risk of thyroid dysfunction in children conceived by IVF. These observations suggest that a high E_2_ environment during the first trimester is directly responsible for the altered thyroid hormone profile in offspring conceived by fresh ET and evaluation of serum E_2_ before ET should be adopted to reduce the possibility of high E_2_ exposure to the developing embryo. Our findings provide new insights into the safety of IVF children and underline the importance of continuous monitoring of endocrine axes of IVF children. Further studies are needed to confirm these findings and to better determine their etiopathogenesis and clinical significance.

## Abbreviations

ART: Assisted reproduction technology
COH: Controlled ovarian hyperstimulation
E_2_: Estradiol
ET: Embryo transfer
HPT: Hypothalamus-pituitary-thyroid
IVF: *In vitro* fertilization

## References

[1] Steptoe PC, Edwards RG (1978). Birth after the reimplantation of a human embryo. Lancet.

[2] Chen M, Wu L, Zhao J, Wu F, Davies MJ, Wittert GA, Norman RJ, Robker RL, Heilbronn LK (2014). Altered glucose metabolism in mouse and humans conceived by IVF. Diabetes.

[3] Hu XL, Feng C, Lin XH, Zhong ZX, Zhu YM, Lv PP, Lv M, Meng Y, Zhang D, Lu XE, Jin F, Sheng JZ, Xu J, Huang HF (2014). High maternal serum estradiol environment in the first trimester is associated with the increased risk of small-for-gestational-age birth. J Clin Endocrinol Metab.

[4] Huang HF, Sheng JZ (2014). Gamete and Embryo-Fetal Origins of Adult Diseases.

[5] Kalra SK, Ratcliffe SJ, Coutifaris C, Molinaro T, Barnhart KT (2011). Ovarian stimulation and low birth weight in newborns conceived through in vitro fertilization. Obstet Gynecol.

[6] McDonald SD, Han Z, Mulla S, Murphy KE, Beyene J, Ohlsson A (2009). Preterm birth and low birth weight among in vitro fertilization singletons: a systematic review and meta-analyses. Eur J Obstet Gynecol Reprod Biol.

[7] Ceelen M, van Weissenbruch MM, Vermeiden JP, van Leeuwen FE (2008). Delemarre-van de Waal HA: **Growth and development of children born after in vitro fertilization**. Fertil Steril.

[8] Whybrow PC, Prange AJ, Treadway CR (1969). Mental changes accompanying thyroid gland dysfunction: a reappraisal using objective psychological measurement. Arch Gen Psychiatry.

[9] Neves C, Alves M, Medina JL, Delgado JL (2008). Thyroid diseases, dyslipidemia, and cardiovascular pathology. Rev Port Cardiol.

[10] Lidegaard O, Pinborg A, Andersen AN (2006). Imprinting disorders after assisted reproductive technologies. Curr Opin Obstet Gynecol.

[11] Gracia CR, Morse CB, Chan G, Schilling S, Prewitt M, Sammel MD, Mandel SJ (2012). Thyroid function during controlled ovarian hyperstimulation as part of in vitro fertilization. Fertil Steril.

[12] Mintziori G, Goulis DG, Toulis KA, Venetis CA, Kolibianakis EM, Tarlatzis BC (2011). Thyroid function during ovarian stimulation: a systematic review. Fertil Steril.

[13] Poppe K, Unuane D, D’Haeseleer M, Tournaye H, Schiettecatte J, Haentjens P, Velkeniers B (2011). Thyroid function after controlled ovarian hyperstimulation in women with and without the hyperstimulation syndrome. Fertil Steril.

[14] Santin AP, Furlanetto TW (2011). Role of estrogen in thyroid function and growth regulation. J Thyroid Res.

[15] Trueba SS, Auge J, Mattei G, Etchevers H, Martinovic J, Czernichow P, Vekemans M, Polak M, Attie-Bitach T (2005). PAX8, TITF1, and FOXE1 gene expression patterns during human development: new insights into human thyroid development and thyroid dysgenesis-associated malformations. J Clin Endocrinol Metab.

[16] Sakka SD, Malamitsi-Puchner A, Loutradis D, Chrousos GP, Kanaka-Gantenbein C (2009). Euthyroid hyperthyrotropinemia in children born after in vitro fertilization. J Clin Endocrinol Metab.

[17] Kondapalli LA, Molinaro TA, Sammel MD, Dokras A (2012). A decrease in serum estradiol levels after human chorionic gonadotrophin administration predicts significantly lower clinical pregnancy and live birth rates in in vitro fertilization cycles. Hum Reprod.

[18] Fleten C, Nystad W, Stigum H, Skjaerven R, Lawlor DA, Davey Smith G, Naess O (2012). Parent-offspring body mass index associations in the Norwegian Mother and Child Cohort Study: a family-based approach to studying the role of the intrauterine environment in childhood adiposity. Am J Epidemiol.

[19] Belkacemi L, Nelson DM, Desai M, Ross MG (2010). Maternal undernutrition influences placental-fetal development. Biol Reprod.

[20] Black RE, Victora CG, Walker SP, Bhutta ZA, Christian P, de Onis M, Ezzati M, Grantham-McGregor S, Katz J, Martorell R, Uauy R (2013). Maternal and child undernutrition and overweight in low-income and middle-income countries. Lancet.

[21] Lakshmy R (2013). Metabolic syndrome: role of maternal undernutrition and fetal programming. Rev Endocr Metab Disord.

[22] Ding GL, Wang FF, Shu J, Tian S, Jiang Y, Zhang D, Wang N, Luo Q, Zhang Y, Jin F, Leung PC, Sheng JZ, Huang HF (2012). Transgenerational glucose intolerance with Igf2/H19 epigenetic alterations in mouse islet induced by intrauterine hyperglycemia. Diabetes.

[23] Fetita LS, Sobngwi E, Serradas P, Calvo F, Gautier JF (2006). Consequences of fetal exposure to maternal diabetes in offspring. J Clin Endocrinol Metab.

[24] Fagman H, Nilsson M (2011). Morphogenetics of early thyroid development. J Mol Endocrinol.

[25] Mullur R, Liu YY, Brent GA (2014). Thyroid hormone regulation of metabolism. Physiol Rev.

[26] Fisher DA, Nelson JC, Carlton EI, Wilcox RB (2000). Maturation of human hypothalamic-pituitary-thyroid function and control. Thyroid.

[27] Bale TL, Baram TZ, Brown AS, Goldstein JM, Insel TR, McCarthy MM, Nemeroff CB, Reyes TM, Simerly RB, Susser ES, Nestler EJ (2010). Early life programming and neurodevelopmental disorders. Biol Psychiatry.

[28] Wallingford JB, Niswander LA, Shaw GM, Finnell RH (2013). The continuing challenge of understanding, preventing, and treating neural tube defects. Science.

[29] Aviles M, Gutierrez-Adan A, Coy P (2010). Oviductal secretions: will they be key factors for the future ARTs?. Mol Hum Reprod.

[30] Chason RJ, Csokmay J, Segars JH, DeCherney AH, Armant DR (2011). Environmental and epigenetic effects upon preimplantation embryo metabolism and development. Trends Endocrinol Metab.

[31] Lonergan P, Fair T (2014). The ART of studying early embryo development: progress and challenges in ruminant embryo culture. Theriogenology.

[32] Duntas LH (2002). Thyroid disease and lipids. Thyroid.

[33] Caraccio N, Dardano A, Monzani F (2005). Subclinical thyroid disease and cardiovascular disease. JAMA.

[34] Ceelen M, van Weissenbruch MM, Roos JC, Vermeiden JP, van Leeuwen FE, Delemarre-van de Waal HA (2007). Body composition in children and adolescents born after in vitro fertilization or spontaneous conception. J Clin Endocrinol Metab.

[35] Ceelen M, van Weissenbruch MM, Vermeiden JP, van Leeuwen FE, Delemarre-van de Waal HA (2008). Cardiometabolic differences in children born after in vitro fertilization: follow-up study. J Clin Endocrinol Metab.

[36] Bowdin S, Allen C, Kirby G, Brueton L, Afnan M, Barratt C, Kirkman-Brown J, Harrison R, Maher ER, Reardon W (2007). A survey of assisted reproductive technology births and imprinting disorders. Hum Reprod.

[37] Chiba H, Hiura H, Okae H, Miyauchi N, Sato F, Sato A, Arima T (2013). DNA methylation errors in imprinting disorders and assisted reproductive technology. Pediatr Int.

[38] Hiura H, Okae H, Miyauchi N, Sato F, Sato A, Van De Pette M, John RM, Kagami M, Nakai K, Soejima H, Ogata T, Arima T (2012). Characterization of DNA methylation errors in patients with imprinting disorders conceived by assisted reproduction technologies. Hum Reprod.

[39] Manipalviratn S, DeCherney A, Segars J (2009). Imprinting disorders and assisted reproductive technology. Fertil Steril.

[40] Furlanetto TW, Nguyen LQ, Jameson JL (1999). Estradiol increases proliferation and down-regulates the sodium/iodide symporter gene in FRTL-5 cells. Endocrinology.

[41] Manole D, Schildknecht B, Gosnell B, Adams E, Derwahl M (2001). Estrogen promotes growth of human thyroid tumor cells by different molecular mechanisms. J Clin Endocrinol Metab.

[42] Banu SK, Govindarajulu P, Aruldhas MM (2002). Testosterone and estradiol differentially regulate TSH-induced thyrocyte proliferation in immature and adult rats. Steroids.

[43] Antico-Arciuch VG, Dima M, Liao XH, Refetoff S, Di Cristofano A (2010). Cross-talk between PI3K and estrogen in the mouse thyroid predisposes to the development of follicular carcinomas with a higher incidence in females. Oncogene.

[44] Lima LP, Barros IA, Lisboa PC, Araujo RL, Silva AC, Rosenthal D, Ferreira AC, Carvalho DP (2006). Estrogen effects on thyroid iodide uptake and thyroperoxidase activity in normal and ovariectomized rats. Steroids.

[45] Ho SM, Tang WY, Belmonte de Frausto J, Prins GS (2006). Developmental exposure to estradiol and bisphenol A increases susceptibility to prostate carcinogenesis and epigenetically regulates phosphodiesterase type 4 variant 4. Cancer Res.

